# The Women's international study of long-duration oestrogen after menopause (WISDOM): a randomised controlled trial

**DOI:** 10.1186/1472-6874-7-2

**Published:** 2007-02-26

**Authors:** Madge R Vickers, Jeannett Martin, Tom W Meade, the WISDOM study team

**Affiliations:** 1 Formerly at MRC General Practice Research Framework, Stephenson House, 158–160 North Gower Street, London, UK; 2 Department of Epidemiology and Public Health, London School of Hygiene and Tropical Medicine, Keppel Street, London, UK

## Abstract

**Background:**

At the time of feasibility work and final design of the trial there was no randomised control trial evidence for the long-term risks and benefits of hormone replacement therapy. Observational studies had suggested that long term use of estrogen was likely to be associated, amongst other things, with reduced risks of osteoporosis and ischaemic heart disease and increased risks of breast and endometrial cancer. Concomitant use of progestogens had been shown to protect against endometrial cancer, but there were few data showing how progestogen might affect estrogen actions on other conditions. Disease specific risks from observational studies suggested that, overall, long-term HRT was likely to be beneficial. Several studies showed that mortality from all causes was lower in HRT users than in non-users. Some secondary cardiovascular prevention trials were ongoing but evidence was also required for a range of outcomes in healthy women. The WISDOM trial was designed to compare combined estrogen and progestogen versus placebo, and estrogen alone versus combined estrogen and progestogen. During the development of WISDOM the Women's Health Initiative trial was designed, funded and started in the US.

**Design:**

Randomised, placebo, controlled, trial.

**Methods:**

The trial was set in general practices in the UK (384), Australia (94), and New Zealand (24). In these practices 284175 women aged 50–69 years were registered with 226282 potentially eligible. We sought to randomise 22300 postmenopausal women aged 50 – 69 and treat for ten years. The interventions were: conjugated equine estrogens, 0.625 mg orally daily; conjugated equine estrogens plus medroxyprogesterone acetate 2.5/5.0 mg orally daily; matched placebo. Primary outcome measures were: major cardiovascular disease, osteoporotic fractures, breast cancer and dementia. Secondary outcomes were: other cancers, all cause death, venous thromboembolism and cerebro-vascular disease.

**Results:**

The trial was prematurely closed during recruitment following publication of early results from the Women's Health Initiative. At the time of closure, 56583 had been screened, 8980 entered run-in, and 5694 (26% of target of 22,300) randomised. Those women randomised had received a mean of one year of therapy, mean age was 62.8 years and total follow-up time was 6491 person years.

**Discussion:**

The WISDOM experience leads to some simple messages. The larger a trial is the more simple it needs to be to ensure cost effective and timely delivery. When a trial is very costly and beyond the resources of one country, funders and investigators should make every effort to develop international collaboration with joint funding.

## Background

The Women's International Study of long Duration Oestrogen after Menopause (WISDOM) developed from a joint initiative of the UK Medical Research Council (MRC) and the UK Departments of Health. At the start of the WISDOM trial observational studies had suggested that long term use of estrogen was likely to be associated with a reduced risk of osteoporosis [1] and ischaemic heart disease (IHD) [2] and an increased risk of breast cancer [3] and endometrial cancer [4]. While concomitant use of progestogens could protect against endometrial cancer [4], it was not clear how progestogen might affect the action of estrogen on IHD or other conditions [5-11]. In addition to the main benefits of HRT, observational data suggested that several other conditions might benefit from long-term HRT. These included: stroke [5,6]; cognitive function, Alzheimer's disease and ischaemic vascular dementia [12,13] tooth loss [14] periodontal disease [15] and oral discomfort [16]; osteoarthritis [17,18] colorectal cancer [19,20]; age-related macular degeneration [21] and possibly cataract [22]. There was also evidence of further risks including: increased risk of deep vein thrombosis and pulmonary embolism [23-27]; ovarian cancer [28] and tinnitus [29] and an exacerbation of some autoimmune diseases [30]. The disease specific risks from observational studies suggested that the overall effect of long-term HRT was likely to be beneficial and several studies showed that mortality from all causes was lower in HRT users than in non-users [31,32]. However, it was acknowledged that since observational studies, even when well controlled, may be subject to bias and confounding still not recognised or allowed for and that the true sizes of the effects may differ significantly [33]. The true balance between the risks and benefits of hormone replacement therapy (HRT) could only be ascertained by a randomised controlled trial.

The need for a randomised controlled trial to investigate the long-term effects of (HRT) was first proposed by an MRC ad hoc group in July 1989. Feasibility studies piloting several designs, and overseen by an MRC Working Party, were undertaken by the MRC General Practice Research Framework, starting in the UK in March 1990 and in Ireland and the Netherlands, partly funded by the European Union (EU) Framework Biomedical and Health Research Programme, in 1992. These indicated that a trial would be acceptable to women and their doctors and that recruitment was feasible [9,10,33]. We submitted a protocol for a full scale trial to the MRC in early 1993. The design incorporated comparison of two types of estrogen and two types of progestogen in recently menopausal women and required international co-operation both to recruit and to fund the trial. To reduce costs the MRC Working Party recommended including women from age 50 to 64 years, so that events could be accrued more quickly, and limiting the comparisons to one estrogen and one progestogen. An MRC Trial Development Group was set up to consider ways of refining the protocol, thus reducing the scale and the cost of the proposed trial.

Meanwhile, the US Women's Health Initiative (WHI) trial using the Postmenopausal Estrogen/Progestin Interventions (PEPI) trial [8] as evidence of feasibility was fully funded and began recruitment [35]. The WHI trial recruited women aged 50 to 79 years and compared conjugated equine estrogen (CEE) with placebo in hysterectomised women and CEE plus medroxyprogesterone acetate (MPA) with placebo in women with a uterus.

The MRC Trial Development Group proposed a UK component with minimally acceptable power and international collaboration to recruit a larger sample to achieve higher power. We submitted an application, jointly to MRC and the UK Departments of Health, in November 1993 for the UK component and began discussions with several pharmaceutical companies for trial medication. Wyeth Ayerst US was the only company willing to provide active and placebo medication. This was the same estrogen and progestogen as used in the WHI trial plus matched placebo. It took almost three years for a decision to fund the UK component of WISDOM to be reached because the cost was high and many menopause clinicians opposed the trial, claiming that the cardiovascular benefit was proven, the effect on breast cancer over-estimated and the results with CEE and MPA unlikely to be relevant to clinical practice ten years hence. We submitted a business case for the trial showing that as long as the trial gave a clear answer to the question of balance of risks and benefits costs were justified. MRC conducted an extensive independent international peer review including a separate review looking at the future relevance of results with CEE and MPA. Funding of £21 million for the UK component in 18,000 women was agreed in August 1996. Set up work for the trial in around 400 UK general practices began in 1997 and recruitment in 1999.

An application was made to the EU Framework IV programme for funds for the international arm of WISDOM requesting full cost funding for some European countries plus co-ordination funding for others and, by reciprocal arrangement, for Australia and South Africa. Despite scoring highly on scientific grounds the application was rejected as it was considered that the WHI trial would answer the questions posed. This decision blocked any contribution to WISDOM from Europe. In 2001, some UK funding was transferred to Australia and New Zealand and, supplemented by funding within these countries, international recruitment began in 2001.

### Evidence emerging during conduct of the trial and impact on WISDOM

During the conduct of WISDOM three, randomised, controlled trials reported on the risks and benefits of HRT in postmenopausal women with pre-existing cardiovascular events [36-38]. The HERS trial demonstrated that combined HRT increased the risk of IHD [36], the EVTET trial that estrogen increased recurrence of venous thromboembolism [37] and Viscoli et al that estrogen increased risk of stroke [38].

In June 2002 the arm of the WHI trial in women with a uterus was discontinued early after an average of 5.2 years of follow-up when the results suggested that risks were likely to outweigh benefits. Women taking estrogen and progestogen (combined HRT) had an increased risk of coronary heart disease, stroke, pulmonary embolism and breast cancer, and a decreased risk of hip fracture and colorectal cancer, compared with placebo [39].

The UK MRC reviewed the data from the WHI and other studies that had reported since WISDOM began and considered the case, made by the investigators with the support of the WISDOM Trial Steering and Data Monitoring Committees, to continue WISDOM. In October 2002 MRC decided to close the trial. Funds were made available for closure visits and for some statistical support but, inevitably, key members of the team found other positions before the WISDOM data could be coded and analysed. This has resulted in long delays in the preparation of papers reporting the main findings from WISDOM. These are now nearing completion and it is hoped that the full data set which also contains much cross sectional data of potential interest will be made widely available. This paper describes the protocol for the trial, the difficulties encountered in recruiting at the expected rate and changes made to increase the rate of recruitment.

### Evidence emerging since the closure of WISDOM

The WHI reported that combined HRT did not appear to protect against development of dementia in women who entered the trial over age 65 years [40] and had no clinically meaningful effect on health related quality of life in postmenopausal women [41]. The WHI trial in hysterectomised women randomised to estrogen or placebo was closed prematurely [42] after an average of 6.8 years of follow up. Women taking estrogen had an increased risk of stroke compared with those taking placebo but neither cardio-protection nor increased breast cancer, had been, or were considered likely to be, found.

The WHI data on breast cancer in women taking estrogen appears to conflict with observational studies suggesting that both combined and estrogen only HRT increase breast cancer in a general population. The observational studies do however report that the increased risk is greater for combined HRT [43,44] and that the effects of HRT are smaller in obese than in thinner women [43]. Since over half the women in the WHI estrogen trial were obese this may account for the lack of effect on breast cancer.

The trial results emerging since WISDOM began and data from large observational studies [44-46] have led to revised guidelines on the use of HRT [47-49] which is now recommended only for short term use to relieve menopausal symptoms. However some have questioned whether the results in the populations studied can be generally applied to recently postmenopausal women [50]. The WHI trial recruited postmenopausal women aged 50 to 79 years, mean 63.3 years, and thus largely reflects the long-term effects of HRT in older women [35]. The women in the WHI trial, particularly in the estrogen only arm, were also, more overweight than average, compared with the US national population figures and some already had documented cardiovascular events whilst amongst many others there was a high prevalence of established cardiovascular risk factors at entry [50]. The other trials reporting effects of HRT on cardiovascular disease were secondary prevention trials [36-38]. A meta-analysis of 30 randomised controlled trials conducted between 1966 and 2002 assessed mortality associated with HRT by age above or below 60 at trial entry. Compared with placebo a significant reduction in mortality was seen in those initiating HRT under age 60 but not in those initiating HRT after age 60 [51].

### Study objectives

The aim of the WISDOM trial was to contribute to establishing the long-term balance between benefits and risks of hormone replacement therapy (HRT). The design allowed for two comparisons: between HRT, mainly combined HRT, and placebo; and, in hysterectomised women only, between estrogen alone and combined HRT.

The main objectives were to estimate the effects of HRT on the incidence of:

• major cardiovascular disease;

• osteoporotic fractures;

• breast cancer;

• dementia; and

• the short and long term effects on quality of life and psychological well-being.

Secondary objectives were to estimate: the effect of HRT on stroke, venous thrombosis, depression, osteoarthritis, other cancers including colorectal and ovarian cancer, age-related macular degeneration, cataract, retinal vein occlusion, dry eye syndrome, tooth loss, periodontal disease, auto-immune diseases and tinnitus; and the health economic implications of HRT usage.

## Methods

### Trial design

**Figure 1:**
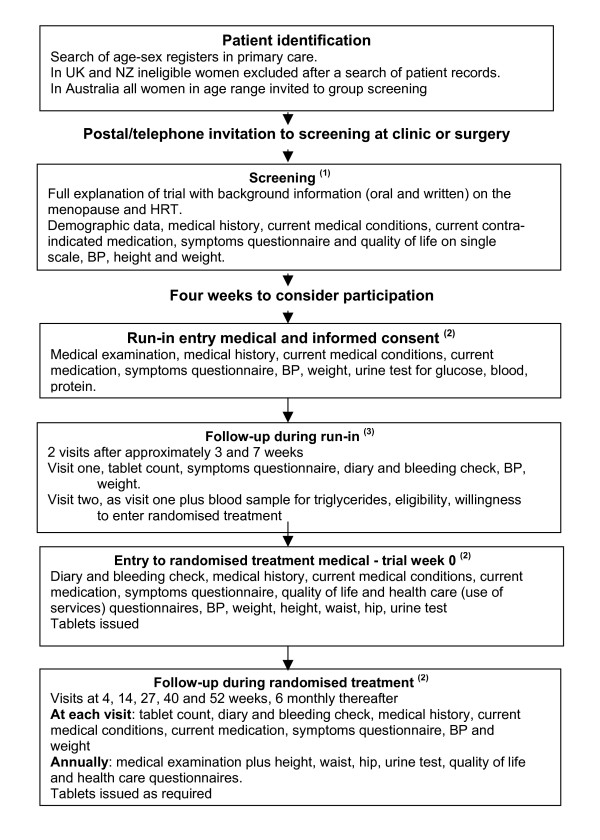
**Flowchart of trial procedures in WISDOM**. ^(1) ^In UK explanation given on individual basis. In NZ and Australia group sessions held and individual interviews held later with women who indicated interest in the trial. ^(2) ^In Australia tablets not held or issued by individual general practices. Participants collected trial medication from the co-ordinating centre's pharmacy. ^(3) ^In Australia blood samples taken at co-ordinating centre and results sent to GP.

WISDOM was designed as a double blind randomised placebo controlled trial. The design allowed for two comparisons: the main comparison between HRT, mainly combined HRT, and placebo; and in hysterectomised women only, between estrogen alone and combined HRT. The different stages of the trial from identification of potential participants through to the closure visit are summarised in Figure 1 and the data collected at the different stages is outlined in Additional file 1.

### Setting

The trial was undertaken in general practices in the UK, Australia and New Zealand. The co-ordinating centre of the UK Medical Research Council (MRC) General Practice Research Framework (GPRF) in London was responsible for overall development and co-ordination.

In the UK, recruitment was nationwide through 384 group general practices that were part of the MRC GPRF. In Australia recruitment was from 91 general practices in three states with overall co-ordination from the Department of Obstetrics and Gynaecology, University of Adelaide and satellite centres at the Faculty of Health at the University of Newcastle and the Baker Medical Research Institute in Melbourne. In New Zealand recruitment was from 24 general practices in three areas with overall co-ordination from the Department of General Practice, Wellington School of Medicine and Human Health and satellite centres in Auckland and Christchurch.

In each general practice one clinician was responsible for the day-to-day conduct of the trial though individual GPs retained ongoing clinical responsibility for their own patients.

### Participants

Postmenopausal women aged 50 to 69 years at randomisation were eligible for the trial. Postmenopausal was defined as no menstrual period in the last twelve months or having had a hysterectomy. Women who had started taking HRT before their menstrual periods had stopped were considered eligible when they had been taking HRT for 12 months.

The exclusion criteria for randomisation were:

• For the placebo controlled components: oral or transdermal HRT use in the last six months, ever use of HRT implant in women with a uterus, HRT implant inserted in last eight months in women with a hysterectomy.

• History of endometriosis (hysterectomised or non-hysterectomised woman) or endometrial hyperplasia in a woman with a uterus.

• History of invasive breast cancer, lobular carcinoma in-situ (LCIS), ductal carcinoma in-situ (DCIS), Paget's disease of the nipple or atypical hyperplasia of the breast.

• Known BRCA1 or BRCA2 mutation carrier.

• History of melanoma.

• Invasive cancer at any other site apart from basal and squamous cell skin cancer within the last 10 years.

• History of meningioma.

• Myocardial infarction, cerebrovascular accident, sub-arachnoid haemorrhage or transient ischaemic attack within the last six months.

• History of currently active liver disease or chronic liver disease but excluding Hepatitis A unless currently active.

• Evidence of severe current renal impairment.

• History of gall bladder disease in a woman who had not had a cholecystectomy or of gallstones following a cholecystectomy.

• History of deep vein thrombosis, pulmonary embolism or retinal vein occlusion.

• Positive thrombophilia screen (Factor V Leiden or prothrombin mutations, Protein C, Protein S or anti-thrombin III deficiencies, APC resistance, dysfibrinogenaemia or antiphospholipid antibodies).

• Otosclerosis.

• Porphyria.

• History of hepatitis B, hepatitis C or HIV (not an exclusion in New Zealand).

• Currently pregnant.

• Currently taking or has taken contraceptive drugs in the last 12 months.

• Current triglyceride level (fasting) > 5.5 mmol/l.

• Active participant in any other intervention trial likely to affect trial outcomes.

• Taking tamoxifen, toremifene, raloxifene or any other selective oestrogen receptor modulator (SERM).

• Other conditions/circumstances where the general practitioner judged that it would not be possible to obtain fully informed consent and/or successfully complete trial procedures.

### Identification of potential participants

Women aged 49–69 years were identified from the general practice registers (Figure 1). Identifying women aged 49 years allowed a maximum of one year for contact, screening and a run-in phase prior to randomisation at 50. The women were assigned WISDOM study numbers and, where possible, their medical records were searched to identify any recorded evidence of ineligibility and factors in the medical history potentially associated with trial outcomes. These searches were conducted by practice nurses and, in data passed to the researchers, potential participants were identified only by study number. The information collected is listed in Additional file 1.

One reason for collecting the medical history of all women in the eligible age range was to be able to assess the generalisability of the results of the trial in the randomised population to that of the registered population. While this was valuable, it was time consuming, and affected the rate of recruitment between 1999 and 2001. The randomisation stage of the trial was not started until January 2001. In July 2001, once sufficient data on which to comment on generalisability had been obtained from the eligible population, the full note search of every woman eligible for the trial was replaced by a quicker eligibility check. From this point the trial nurses checked the patients' medical records against a list of the ineligibility criteria and recorded reasons for ineligibility.

### Screening

In the UK, starting with the older age band first, those who were potentially eligible for the trial were contacted by a letter from their general practitioner and invited to attend a screening session with a research nurse at their practice, The women were told that the practice was taking part in a large study on the health of post-menopausal women that included a long-term trial of HRT. They were invited to the surgery to discuss their health, the menopause, the benefits and risks of HRT, the trial and their possible participation in the trial. If the women preferred, these screening interviews could also be conducted in their homes.

In Australia, patients were first invited to group sessions to learn about HRT and the trial while in the UK and New Zealand these aspects were covered as the first part of the screening interview. Many women attended for a screening interview from general interest and to discuss their health, but had no intention of entering a trial; others were initially interested but once they were given more information about the trial, decided not to participate. This was another factor affecting recruitment. From May 2002 the patient information leaflet was included with the invitation letter from the GP, giving more information about the trial so that women who definitely had no intention of participating were less likely to attend a screening interview. This increased the rate at which those likely to participate could be screened and recruited.

**Figure 2:**
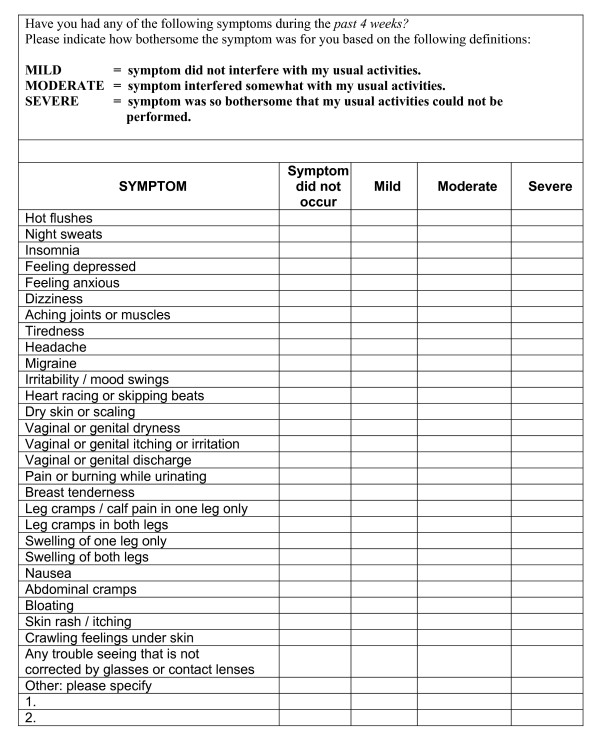
WISDOM trial symptoms questionnaire.

At the screening interview women completed a nurse administered screening questionnaire covering demography, medical history, including trial exclusions and known risk factors for the trial end-points, current medical conditions, contra-indicated medication, symptoms (Figure 2), recent cervical screening and mammography and overall quality of life. Blood pressure, height and weight were measured. From May 2002, to reduce the time taken to conduct a screening interview, height and weight were not measured and details of ethnic origin were not collected. Collection of full information on lifestyle, fractures and current medical conditions was deferred until the randomisation visit.

Any discrepancies between data previously extracted from the patient's notes and the patient's recall were identified and highlighted by the computerised patient management program so that the nurse could resolve them, if necessary by consulting the GP. Data collected are listed in Additional file 1.

Potential participants were given a full explanation of the menopause, current knowledge of the risks and benefits of HRT, the rationale for the trial, details of the trial including all procedures involved and visits required. The explanatory materials were developed in collaboration with a lay panel of women.

**Figure 3:**
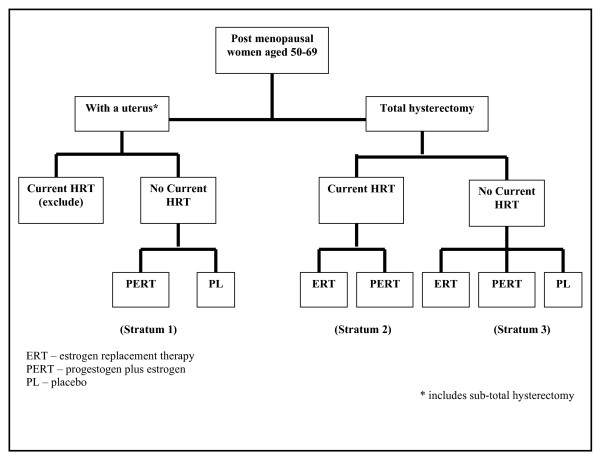
Treatment allocation in the WISDOM trial.

Those women eligible on the basis of the questionnaires were invited to consider participating in the trial. Women with an intact uterus or sub-total hysterectomy, taking HRT and unwilling to discontinue for a washout period before randomisation, were excluded. Other potentially interested women were told they would be assigned to one of three strata (figure 3):

**1. **Women with an intact uterus or sub-total hysterectomy not taking HRT were assigned to stratum one and randomised to combined estrogen and progestogen replacement therapy or placebo.

**2. **Hysterectomised women taking, and wishing to continue taking, HRT were assigned to stratum two and randomised to estrogen only HRT or combined HRT.

**3. **Hysterectomised women not taking HRT were assigned to stratum three and randomised to estrogen only HRT or combined HRT or placebo.

They were given clear written explanation of the main points covered in the interview and an appointment was arranged two to four weeks later for a medical examination to enter the run-in phase of the trial and were asked to cancel this if they decided not to proceed. All women were asked for their written consent for their medical and national records to be used in the future and for these records and their trial data to be passed to the researchers.

### Run-in

To ensure that they were fully committed to the trial before randomisation and had no reaction to the excipients in the trial medication, participants were asked to complete a run-in period of approximately twelve weeks. At the entry to run-in visit the nurse recorded symptoms, blood pressure, weight, urinary protein, glucose and blood and checked that the woman remained eligible for the trial (see Additional file 1). The GP conducted a general medical examination and made the decision on eligibility. The nurse ensured that the procedures for the trial and possible risks and benefits were understood by the women and that all questions had been answered.

Those willing to enter the trial gave written informed consent and were issued with tablets for the run-in phase. All women took medication during the run-in: women who were eligible for the placebo controlled randomisation (strata 1 and 3) took placebo; those who were not (stratum 3) took estrogen only HRT.

Participation in the National Breast Screening Programmes was encouraged but was not mandatory; advice and information on breast awareness was given. Women with a uterus were given diaries to record any vaginal bleeding, concurrent medication and illnesses. All patients received a card stating that they were in the trial with contact details for the practice, the co-ordinating centre and, for use by medical staff, the telephone number for the 24 hour emergency code break.

There were two visits to the surgery during the run-in period, at approximately three and eight weeks after entry, mainly to check on tablet compliance and ensure that the woman remained eligible (Table 1). At the second run-in visit, those reaching 80% compliance were invited to enter the randomised phase of the trial. If patients were willing to enter but had not reached 80% compliance one further run-in was permitted.

In the UK these women were asked to give a blood sample to assess the last criterion for eligibility, a fasting triglyceride level < 5.5 mmol/l, and to provide samples for future analyses. A 25 ml non-fasting blood sample was taken initially: 5 ml for immediate estimation of triglyceride and cholesterol levels and 20 ml to store as serum, red blood cells, buffy coat and plasma for future analyses. Samples were prepared at the practices and posted to arrive within 24 hours at the trial co-ordinating centre for analysis. Patients with a non-fasting triglyceride level > 5.5 mmol/l were recalled for a second triglyceride measurement following an overnight fast. Patients with fasting triglyceride levels ≥ 5.5 mmol/l were excluded from the trial and advised to consult their GP. In Australia and New Zealand, this criterion for eligibility was waived. Women who were unwilling to give a blood sample in the UK could enter the trial without a blood sample if their GP had agreed eligibility.

### Randomisation

After the second run-in visit women who were eligible and willing to participate were randomised centrally and appropriate medication dispatched to the practice in time for the baseline randomisation visit. Participants were assigned to treatment in accordance with the schema at Figure 3 using a computer based, stratified block randomisation program to ensure that within each stratum the treatment groups were of virtually equal size. The stratification was based on hysterectomy status and HRT use at the time of randomisation. In stratum one, women with a uterus were randomised to combined HRT therapy using a block size of 16. In stratum two, hysterectomised women who were only prepared to take active treatment were randomised to estrogen only HRT or combined HRT using a block size of 16. In stratum three, hysterectomised women were randomised to estrogen only HRT, combined HRT or placebo using a block size of 24.

The baseline questionnaire at the randomisation visit (see Additional file 1) covered risk factors, possible adverse events, all outcomes, quality of life and other medical history to check that they remained eligible. Use of health services and details of the severity of certain conditions eg, systemic lupus erythematosus were obtained from the patients' notes. The nurse measured height, weight, and waist and hip circumference using standard operating procedures and the GP then made a final decision on eligibility. A patient was recorded as entering randomised treatment if she left the surgery with her first pack of randomised treatment tablets.

### Blinding and protection from bias

As far as possible, the trial was conducted in a double-blind manner. The treatment assignment was directly accessible only to the computer officer responsible for the randomisation, the trial statisticians and the staff supplying a 24 hour code break service. Computer generated lists were sent to the drug dispensing manager along with patient labels. Active and placebo medications were matched for size, colour and taste. Within each stratum labelling and packaging was identical and randomly generated tablet identification codes were used so that the participant, the nurse and the GP were blinded to the treatment allocation. No-one involved with assessment of outcomes was aware of treatment assignment. During the trial it was not possible to maintain full blindness as reports of vaginal bleeding or spotting triggered a code break and an investigation to ensure that any abnormal bleeding was not overlooked.

### Interventions

Estrogen-only HRT was conjugated equine estrogens (Premarin, Wyeth Ayerst), 0.625 mg orally daily. Combined progestogen and estrogen HRT was conjugated equine oestrogens as above plus medroxyprogesterone acetate (MPA) 2.5 mg orally daily (Prempro, Wyeth Ayerst US). Women with a uterus within three years of their last period, or aged 50–53 years, or aged 54 or over with unacceptable breakthrough bleeding took a higher dose of 5.0 mg MPA (Premique, Wyeth Ayerst). Women who experienced unacceptable intermittent spotting or bleeding with combined HRT containing 5.0 mg MPA were offered open label Premique cycle (Premarin 0.625 mg orally daily plus MPA 10 mg orally for the last 14 days of a 28 day cycle). Women could choose to remain on Premique cycle for the remainder of the trial or could return to their continuous combined treatment. Estrogen-only HRT, combined HRT and matched placebo containing the same excipients were prepared and supplied by Wyeth Ayerst US.

### Follow-up

It was planned to recruit women over a three year period and to end the trial nine years after the last participant was randomised giving a median of 10.5 years follow up, and a maximum of 12 years. Women were to be seen at their general practice at 4, 14, 27, 40 and 52 weeks and then 6 monthly. Nurses contacted participants between visits, by telephone, to provide advice and support as required. A final visit was to take place as soon as possible after the closure of the trial.

Because of the decision in October 2002 to close the trial early when the WHI results indicated that the likely balance of risks and benefits of HRTwas unfavourable the median follow up time for WISDOM was 11.9 months (IQR: 7.1 months to 19.6 months).

Patient compliance was assessed at each visit by tablet count. At the annual visit information was collected on risk factors, possible adverse events, all outcomes and on other medical history to check that patients remained eligible (see Additional file 1). At other visits information was collected on risk factors, possible adverse events, primary and secondary outcomes and on other medical history to check that patients remained eligible (see Additional file 1). There was provision for recording information received by the nurse or the GP from the patient, and with the patient's permission, from a friend or a relative between follow-up visits. A further check of general practice medical records was made annually.

Outcomes were assessed at face to face interviews between the patient and the nurse, using validated questionnaires wherever possible, and entering data directly onto laptop computers using a suite of software specially designed for the trial which included a real time patient management system in accordance with the protocol. Electronic data checking at the time of collection incorporated extensive checks for plausibility, consistency and completeness. Data were sent to the co-ordinating centre within one week of collection by modem link within the UK and via a standard File Transfer Program server site from Australia and New Zealand. A member of the study team, who was blinded to treatment allocation, obtained any data needed to confirm a clinical event from the GP, hospital or coroner.

It had been planned to ascertain fatal end-points and cancer registrations in the UK via the National Health Service Central Register, in Australia via the National Death Index database and in New Zealand via National Health Index number tracing, death registrations, hospital records and the New Zealand Cancer register but with the early closure this was not pursued.

To fully assess the long-term effects of HRT and to investigate changes following discontinuation of treatment, follow up by medical records in the general practice and national records had been planned for a further five years after treatment but following closure these plans were abandoned.

### Outcomes

The primary outcomes were:

• major cardiovascular disease, defined as the sum of one or more of fatal and non-fatal myocardial infarction, unstable, hospitalised angina and sudden coronary death;

• osteoporotic fractures (hip, wrist and all fractures other than skull, face, cervical spine, fingers and toes);

• incidence of breast cancer; and

• quality of life and psychological well-being;

Secondary outcomes were:

• breast cancer mortality;

• other cancers, incidence and mortality;

• venous thromboembolism (deep vein thrombosis, pulmonary embolism, retinal vein occlusion; and

• cerebro-vascular disease.

The following conditions were also investigated: depression; incontinence; symptomatic arthritis; tooth loss; periodontal disease; tinnitus; asthma; low back pain; age-related macular degeneration; cataract; and dry eye syndrome. Use of health services was recorded to allow an estimate of the health economic implications of HRT usage.

### Health economic analysis

It had been planned to evaluate the health economic implications of HRT by estimating, and modelling where necessary, costs of treatment, costs of conditions prevented or caused by HRT, costs of all use of health care and indirect costs such as time lost from work/usual activities and social security implications. Overall cost effectiveness was to be calculated as costs per Quality Adjusted Life Year (QALY) gained using the quality of life measured by the EuroQol. In the light of the early closure this analysis did not proceed.

### Dementia and cognitive decline sub-study

In an add-on study in sub-samples of WISDOM participants, the effects of HRT on the incidence and progression of dementia and on the onset and progression of cognitive decline were assessed. This work was led by Professor Martin Prince (Institute of Psychiatry, London), Professor Felicia Huppert (Psychiatry, Cambridge) and Dr Marcus Richards (University College, London) and will be described in a separate publication.

### Confirmation of outcomes

Primary and secondary clinical outcomes were reviewed blind to treatment allocation. For cardiovascular disease, cerebrovascular disease, venous thromboembolism and cancers this was done by an independent assessor. For fractures the assessment was done by a member of the study team with a 10% sample reviewed by an independent assessor.

### Adverse events

An adverse event was defined as any untoward medical occurrence regardless of whether a causal relationship with trial treatment was suspected. The event was classified as serious if it was life-threatening, required inpatient hospitalisation or prolongation of existing hospitalisation, or caused significant or persistent disability or incapacity. An unexpected adverse event was an experience not previously reported (in nature, severity or incidence) in connection with the trial treatment. For confirmed serious adverse events which were trial exclusions, the protocol required either a permanent or a temporary interruption of trial treatment (see Additional file1). Where the protocol did not specify a course of action the nurse informed the GP and the trial clinician by memo and they liaised to decide what action to take. Whenever possible, the GP and clinician remained blind to treatment allocation. For minor adverse events, for example those recorded on the symptoms questionnaire, the nurse and, if necessary, the GP, advised palliative treatments, using guidelines, with the aim of keeping the patient in the trial on their randomised treatment. New onset migraine and current thrombophlebitis triggered a temporary interruption of treatment.

### Vaginal bleeding in women with a uterus

Intermittent vaginal bleeding was expected as a temporary side effect of continuous combined HRT in some women with a uterus randomised to PERT. Bleeding in women with a uterus was monitored by asking the women to complete a daily diary noting episodes of bleeding according to the following categories:

• spotting – spotting/streaking not requiring sanitary protection;

• light – small amount of bleeding requiring some protection;

• moderate – less than a normal period;

• severe – normal period or heavier;

and recording any concurrent medical conditions, medication and whether trial medication had been missed.

The diaries were monitored at scheduled visits and between scheduled visits women were asked to telephone the trial nurse to report bleeding. The trial nurse recorded occurrence of bleeding, severity, duration and whether bleeding was continuous or intermittent.

At the beginning of the trial, best practice advice suggested that to ensure that pathological bleeding was not overlooked, all reports of vaginal bleeding continuing for at least four weeks (continuous or intermittent) or bleeding over a shorter period which was considered to be severe, continuous or rapidly worsening, should trigger a request by the trial nurse for a break in the treatment code. Those taking placebo would then be referred to the GP, those taking combined HRT with 2.5 mg MPA offered 5.0 mg MPA, and those already taking 5.0 mg MPA asked to continue taking trial treatment for a further six weeks. Then, if bleeding continued, participants would be referred to the GP for investigation. If nothing abnormal were found, participants would be offered second line treatment with Premique Cycle. Women reporting only spotting would be monitored without a code break and the advice of the GP sought. This approach resulted in many women withdrawing themselves from randomised treatment as they were not prepared to wait for a protocol code break.

In January 2001, after consulting with the Steering and Data Monitoring Committees, procedures for the management of bleeding were revised to reduce withdrawals from the trial

To ensure that pathological bleeding was not overlooked, all reports of vaginal bleeding, including spotting, triggered an immediate break in treatment code. Patients were asked to continue on trial medication during an investigation of bleeding.

• Bleeding that started on placebo treatment

Women randomised to placebo were referred immediately to their GP for investigation in line with their usual clinical practice.

• Bleeding that started on active treatment

The management of women on active treatment depended on when bleeding occurred relative to treatment and previous amenorrhoea.

• Bleeding that started within 90 days on active treatment.

Vaginal bleeding is very common in the first few months on continuous combined hormone replacement therapy but usually resolves. Women with bleeding starting in the first 90 days of active treatment (excluding any treatment interruptions) were referred to their GP only if they or their nurse were concerned. Women taking 2.5 mg MPA were offered a dose increase to 5 mg as soon as bleeding was reported. Women already on 5 mg MPA had no change to their medication. Patients were monitored by the nurse for 90 days from the date bleeding started. Any woman who experienced vaginal bleeding after the 90-day monitoring period was referred to her GP.

It was recommended that women whose dose of MPA was increased to 5 mg because of bleeding in the first 90 days remained on the higher dose until 3 months amenorrhoea had been achieved. Thereafter the dose could be reduced to 2.5 mg, but should bleeding reoccur within 21 days of the dose reduction, it was recommended that the patient resume taking 5 mg MPA.

• Bleeding that started after 90 days amenorrhoea on active treatment.

Women with bleeding that started after 90 days of amenorrhoea on active treatment were referred immediately to their GP unless the bleeding occurred within 21 days of a treatment interruption, reduction in the dose of MPA from 5 mg to 2.5 mg or following diarrhoea, vomiting or a course of antibiotics. If one of these possible reasons applied, the patient was monitored by the nurse and referred to her GP if bleeding continued beyond 21 days.

• Persistent bleeding

Women with persistent bleeding for which no pathological cause was found were offered second-line treatment using Premique Cycle. Women with a uterus taking second-line treatment who reported bleeding which was heavier than their monthly menstrual bleed had been pre-menopause or of longer duration were referred to the GP for investigation.

### Vaginal bleeding in women with a hysterectomy

Women with a hysterectomy (sub-total or total) reporting vaginal bleeding were referred immediately to their GP for investigation.

### Discontinuation of trial treatment

Permanent discontinuation of trial treatment was required if any of the exclusion criteria for the trial were confirmed during follow up. If participants developed symptomatic or active gall bladder disease a temporary interruption of treatment was made and if the participant subsequently had a cholecystectomy they were invited to recommence trial treatment. A temporary interruption of treatment was required to investigate sudden loss of vision, onset of proptosis or diplopia.

When trial outcomes occurred that were not trial exclusions (myocardial infarction, unstable hospitalised angina pectoris, cerebrovascular accident, subarachnoid haemorrhage, TIA, osteoporotic fractures) participants were able to remain on their allocated treatment to contribute to other outcomes. However, a temporary interruption of treatment could be initiated at the discretion of the GP or other clinician or by the participant. To reduce the risk of venous thromboembolism, a temporary interruption could be made during hospitalisation and following accidents resulting in immobilisation. Trial treatment could also be discontinued at the discretion of the GP for symptoms possibly associated with trial treatment, other medical or non-medical reasons. Participants were free to initiate withdrawal from the trial or temporary interruption of treatment without giving a reason.

GPs and participants were encouraged to try a temporary interruption of treatment first and were requested to discontinue treatment permanently only after consultation with the trial clinician and after all efforts had been made to keep the patient on randomised treatment.

### Minimising loss to follow up

All patients who discontinued trial treatment were followed up and every effort was made to contact patients after non-attendance at follow up appointments to minimise losses to follow up. Where participants did not attend for visits, but had given permission for their medical records to be accessed, annual and final searches were undertaken.

### Sample size and comparisons

The original minimum sample size for WISDOM, set at the time of the grant application, was 18000, to detect, with 80% power at the 5% level of significance, a 25% decrease in ischaemic heart disease (excluding unstable angina) and stroke over ten years comparing, in women aged 50–64 years at randomisation, those randomised to HRT (combined HRT plus estrogen only HRT) with those randomised to placebo. It was agreed that an actual recruitment target of 23000, to include recruitment from countries other than the UK, was feasible.

The primary outcome was subsequently changed to exclude stroke and include unstable angina and the age range was extended up to 69 years. Other assumptions were modified reflecting falling coronary event rates in the general population, new information from the Women's Health Initiative (personal communication to MRC WISDOM Steering Group) on likely times to treatment effects and fall off in treatment effects after discontinuation of treatment, and revised estimates for withdrawal from randomised treatment. Sample size calculations were revised to calculate what might be detected with a sample of 23000. Comparing combined HRT with placebo it was estimated that WISDOM would have 80% power at the 5% level of significance to detect at ten years:

• for cardiovascular disease (including unstable angina), a 29% reduction from a placebo event rate of 39/1000;

• for all osteoporotic fractures, a 20% reduction from a placebo event rate of 95/1000; and

• for breast cancer, a 40% increase from a placebo event rate of 36/1000.

For cardiovascular disease calculation of event rates in the trial population not receiving active treatment incorporated the following.

• Over the period 1997 to 2010 an assumed reduction in mortality from IHD of 40% and drop in incidence rate of 20%.

• Withdrawal from randomised treatment by 10% of participants in the first year of treatment, 7% in year 2, 4% in year 3, 3% in year 4 and 2% in years 5 – 10, giving a total withdrawal at some stage of 36%. An approximation to the change in the event rate observed can be made assuming 70% of HRT group have been on treatment for full 10 years and 30% have no treatment.

• For IHD 2.5 non-fatal events occurring for each fatal event as suggested by the MONICA study, total numbers (i.e. fatal plus non-fatal) then being derived from ONS age-specific mortality rates in 1997.

• In the absence of reliable estimates of the incidence of unstable angina for women in the relevant age group, the best estimate was a rate of about 1 per 1000 women per year. Over the ten years of the trial this rate would generate 9.7 unstable angina events per 1000 women entered. However, it was estimated that 50% of these would lead to myocardial infarction, giving an incidence of independent unstable angina events of 4.8 per 1000 women.

• A healthy volunteer effect leading to event rates only 67% of those based on the sum of fatal and non-fatal episodes in the general population.

• Based on feasibility studies and current use of and interest in HRT 20% of the trial population being in the age range 65–69 years and 80% being distributed equally across the three five year age bands, 50–54, 55–59, 60–64 years.

• A 3 year recruitment, which would give 33% of women in study for 11 years, 33% in study for 10 years and 33% of women in study for 9 years.

Using these assumptions the average probability of a major cardiovascular event within ten years in women in the age group 50–69 was estimated to be 0.039.

For the main comparison of HRT versus no HRT, only women who could be randomised to receive placebo could contribute (strata 1 and 3, Figure 3). Based on feasibility studies the proportions expected in the different strata were: stratum 1, 58% equally divided between combined HRT and placebo; stratum 2, 22% equally divided between combined HRT and estrogen-only HRT; stratum 3, 20% equally divided between combined HRT, estrogen-only HRT and placebo. The majority of women on HRT (~84%) would be on combined HRT. Of the total sample size, 78% would be used to compare HRT with placebo (the remaining 22% contributing to the ERT v PERT comparison). The sample sizes have been calculated to achieve significance in a combined HRT versus no HRT comparison, with equal numbers of women randomised to combined HRT and placebo. The target sample size of 22300 gives statistical power of 80% (at 5% level of significance) to detect a reduction of 29% in the cardiovascular end-point. Power for an HRT (combined HRT plus estrogen only HRT) versus no HRT comparison would be slightly increased by the inclusion of hysterectomised patients (within stratum 3) randomised to estrogen-only HRT.

A 29% reduction in risk in those taking HRT would reduce the average probability of a cardiovascular event to 0.028, an absolute reduction of 0.011. If this was achieved then treating 89 women for ten years with HRT would prevent one cardiovascular event. Allowing for withdrawals as described above the average probability of an event would be 0.031 and an intention to treat 125 women for ten years with HRT would prevent one cardiovascular event.

For a direct comparison of estrogen only HRT with combined HRT, approximately 8,000 women could contribute: those in stratum 2 together with those in stratum 3 randomised to HRT. With equal numbers being randomised to estrogen only HRT and combined HRT a 45% difference in cardiovascular events between the groups could be detected with statistical power of 80% (at 5% level of significance).

For the osteoporotic fracture and cancer outcomes it was assumed that the final assessment would be made five years after the end of treatment. No assumptions were made about HRT use following the end of the trial.

In estimating the trial event rate for fractures it was assumed there would be no change in the current fracture rate until 2010. Incidence rates for hip fracture were estimated using figures from the European Report on Osteoporosis, 1998. No trend figures were available and no trend was assumed. Rates were reduced by 20% for a healthy volunteer effect. At the end of the 5 year post treatment follow-up period, 27 hip fractures per 1000 women recruited would be expected. Based on published data the risk ratio was estimated as 0.7 in the first year on treatment, 0.6 for second year, 0.5 for years 3 – 10, gradually increasing back to 1 after treatment ends. This would give an expected reduction in events of 26% from 27 per 1000, to 20 per 1000 women recruited. The sample size of 23000 would be able to detect only a larger reduction in hip fractures of 36% with 80% power at the 5% level of significance. We therefore looked at all osteoporotic fractures. As there were no UK incidence rates available for all osteoporotic fractures we assumed the same ratio between hip and all osteoporotic fractures as in US. Rates were reduced by 20% for a healthy volunteer effect. At the end of 5 year post treatment follow-up period, 95 fractures per 1000 women recruited would be expected. Assuming the risk ratio for all fractures was similar to that for hip fractures a 28% decrease in event rate at 5 year follow-up would be expected. A sample size of 23000 could detect a smaller reduction of 20% for all osteoporotic fractures.

The incidence rate for breast cancer rose by about 50% in those aged 50 -69 years over the period 1982–1992. In estimating the trial event rate for breast cancer it was assumed that no further increase would occur. Incidence rates were adjusted down by 10% to allow for the fact that those with near relatives having had breast cancer were unlikely to enter the trial. No other healthy volunteer effect was allowed for. With these assumptions, the expected trial event rate for breast cancer at the end of the 5 year post treatment follow-up period was 36 per 1000 women entered. With a sample size of 23000 it would be possible to detect a 40% increase from a placebo event rate of 36/1000 women-years. This was much higher than the expected increased risk; observational studies suggested that the risk ratio increased by 2.3% for each year on treatment and reduced by the same amount after treatment ends. This would give an expected 11% increase in event rate at 5 years follow-up, an increase from 36 per 1000 women to 40 per 1000 women recruited. It was thus anticipated that WISDOM data would be most appropriately used to contribute to a meta-analysis of the effect of HRT on breast cancer.

The incidence rates for colorectal, ovarian, cervical and endometrial cancers are low and WISDOM would be able to detect only very large increases/decreases associated with HRT use. Estimated event rates for other cancers at 5 year post treatment follow-up were: colorectal 19 per 1000 women recruited; ovarian 8 per 1000 women recruited; cervical 3 per 1000 women recruited; and endometrial 6 per 1000 women recruited.

### Statistical methods

Follow-up time will be calculated for each participant from date of randomisation until the first among: date of outcome, date of death, or 22 October 2002 (when the trial was formally halted). The intention to treat principle will be adopted when assessing treatment effects, participants being classified according to randomisation group and analyses being made comparing HRT (combined HRT plus estrogen only HRT) or combined HRT alone with placebo, and combined HRT with estrogen only HRT. To account for the prospective nature of the data, Kaplan-Meier outcome-free survival curves will be computed and Cox proportional hazard models fitted (after graphically checking the underlying proportional hazards assumptions). The results will be reported as estimated hazard ratios for the effect of HRT versus placebo, combined HRT versus placebo or combined HRT versus ERT, (with 95% confidence intervals), with associated likelihood ratio tests for their significance [62].

## Results

The trial was prematurely closed during recruitment following publication of early results from the Women's Health Initiative. At the time of closure, 56583 had been screened, 8980 entered run-in, and 5694 (26% of target of 22300) randomised. Those women randomised had received a mean of one year of therapy, mean age was 62.8 years and total follow-up time was 6491 person years. The results will be published separately.

## Discussion

The background and history of the WISDOM trial and its early closure have been described elsewhere [63]. It is however fitting to conclude this protocol paper by reflecting on some of the difficulties encountered in setting up a project of this size and complexity and the lessons that may be learned.

WISDOM was beset with delays. From the initial recognition in 1988 that research was needed, to agreement that a randomised controlled trial was the appropriate design, took the UK MRC and Departments of Health two years. A further six years elapsed from the start of the commissioned pilot studies in 1990, to the release of funding for the main trial, despite the demonstration by early 1993 that the trial was feasible in the UK and other European countries. The factors contributing to the delay were understandable concerns about cost, but also opposition by many UK clinicians and scientists working in the menopause area on the grounds that the risk benefit profile of HRT had clearly been shown to be favourable by observational studies. They argued that long-term HRT would reduce osteoporotic fractures and cardiovascular disease and was unlikely to have a significant effect on breast cancer and hence that the funds for the trial would be better spent elsewhere. While the UK was considering ways to reduce or spread costs beyond the UK, and answer the concerns of those opposed to the trial, the WHI trial in the US was conceived, funded and started. Once WHI was in place other countries were no longer prepared to commit funds to WISDOM, despite the successful European feasibility studies and commitment by senior clinicians internationally. Thus the design eventually funded in the UK by MRC and the Departments of Health was an affordable compromise, not the optimum design originally proposed (recently postmenopausal women and two HRT preparations). In the future, when key questions require very costly trials, international collaboration and joint funding by several countries, including the UK and the US, should be pursued. For HRT, had this been possible, the original design for a trial of two types of oestrogen and two cHRT preparations in women aged 50–60 years might have been undertaken. Two important questions which remain unanswered: i) the effect of long-term HRT started close to the menopause and ii) whether there are differences between different types of HRT could then have been addressed.

The set-up and recruitment phase for WISDOM also took longer than anticipated. A decision was made to collect data directly on to laptop computers and download to the co-ordinating centre and to undertake all the design and programming in house. We aimed for technical perfection and the system eventually incorporated a sophisticated real time patient management program and was the prototype for data collection in other trials in the MRC GPRF. This worked extremely well and was one of the success stories of the trial, but it resulted in a tendency to agree to collect data on more and more outcomes of interest for additional sub-studies in order to explore fully the potential risks and benefits of HRT. WISDOM would have progressed more rapidly if we had restricted data collection to the main outcomes and had devolved more of the decisions about individual patient care to the clinicians at the participating sites.

During the six years delay between the application for funding in 1993 and the start of recruitment in 1999 women's use of and perceptions about HRT had changed and UK general practice had been re-organised. This impacted on the trial in that the window of opportunity for rapid recruitment had been lost. Though new strategies were proving successful and have since been used in other trials, recruitment was slower and more costly than anticipated. While it is unlikely that, had WISDOM recruited a greater proportion of its target before the WHI results were published, closure could have been avoided, younger, more recently post-menopausal women would have been included (the strategy was to recruit older age groups first) adding new information not available from the WHI trials.

The WISDOM experience leads to some simple messages. The larger a trial is the more simple it needs to be to ensure cost effective and timely delivery. Investigators should resist gathering data for others, which, while valuable, distracts from the main purpose of the trial. When a trial is very costly and beyond the resources of one country, funders and investigators should make efforts to develop international collaboration with joint funding. If it is not feasible to undertake the optimum design because of lack of funds, investigators should be sure that a compromise design, recommended by funders, really does address the key questions posed.

## Abbreviations

**APC **Activated protein C

**CEE **Conjugated equine estrogen

**ERT **Estrogen replacement therapy

**EU **European Union

**HRT **Hormone replacement therapy

**cHRT **combined estrogen and progestogen HRT

**GPRF **General Practice Research Framework

**IHD **Ischaemic heart disease

**MPA **Medroxyprogesterone acetate

**MRC **Medical Research Council

**PERT **Progestogen and estrogen replacement therapy

**WISDOM **Women's International Study Of long-Duration Oestrogen after Menopause

**WHI **Women's Health Initiative

## Competing interests

The author(s) declare they have no competing interests.

## Authors' contributions

MRV designed the main trial based on feasibility studies, led, was responsible for, the development of the protocol, had overall responsibility for the trial and drafted the paper. JM contributed to the development of the protocol, had responsibility for nursing aspects of the trial and commented on the draft paper. TWM designed the feasibility studies, had clinical responsibility for the trial until October 2001, contributed to the development of the protocol and commented on the draft paper. All authors read and approved the final paper.

## Pre-publication history

The pre-publication history for this paper can be accessed here:



## Supplementary Material

Additional file 1 WISDOM data collection summary. Table summarising information collected at different stages of the WISDOM trial. Click here for file
