# Supplementary material for: The Women's international study of long-duration oestrogen after menopause (WISDOM): a randomised controlled trial
Source: BMC Womens Health. 2007 Feb 26;7:2. doi: 10.1186/1472-6874-7-2 (PMC1828722; doi:10.1186/1472-6874-7-2)
Supplement: Additional file 1 — WISDOM data collection summary. Table summarising information collected at different stages of the WISDOM trial. [file 1472-6874-7-2-S1.doc]

**Additional file 1**

**WISDOM data collection summary**

|  | Notesearch | | Screen | | **Run-in**  **entry** | | Run-in **visit 1** | | Run-in **visit 2** | Randomisation | Follow **up** | Annualmedical |
| --- | --- | --- | --- | --- | --- | --- | --- | --- | --- | --- | --- | --- |
| **Demographic** |  | |  | |  | |  | |  |  |  |  |
| Name |  | | X | | X | | X | | X | X | X | X |
| Address/postcode |  | | X | | X | | X | | X | X | X | X |
| Telephone |  | | X | | X | | X | | X | X | X | X |
| NHS number |  | | X | | X | | X | | X | X | X | X |
| General Practice | X | | X | | X | | X | | X | X | X | X |
| Date of birth | X | | X | | X | | X | | X | X | X | X |
| Marital status |  | | X | |  | |  | |  |  |  |  |
| Education |  | | X | |  | |  | |  |  |  |  |
| Occupation |  | | X | |  | |  | |  |  |  |  |
| Partner’s occupation |  | | X | |  | |  | |  |  |  |  |
| House type |  | | X | |  | |  | |  |  |  |  |
| Car access |  | | X | |  | |  | |  |  |  |  |
| Ethnicity , religion, language** |  | | X | |  | |  | |  |  |  |  |
| Own/parents’ language/religion ** |  | | X | |  | |  | |  |  |  |  |
| Grandparents’ place of birth ** |  | | X | |  | |  | |  |  |  |  |
| Birth weight (patient history) |  | | X | |  | |  | |  |  |  |  |
| Full term/premature |  | | X | |  | |  | |  |  |  |  |
|  | | | | | | | | | | | | |
| **Lifestyle** |  | |  | |  | |  | |  |  |  |  |
| Exercise (frequency & level) * |  | | X | |  | |  | |  | X |  | X |
| Smoking (dates & no.) ** |  | | X | |  | |  | |  |  |  |  |
| Smoking since last asked (type/amount) |  | |  | |  | |  | |  | X |  | X |
| Passive smoking (ever, when, duration) |  | |  | |  | |  | |  | X |  | X |
| Alcohol (type & amount/wk at 20 y, 30y & present) ** |  | | X | |  | |  | |  |  |  |  |
| Alcohol-current (type & amount/wk) ** |  | |  | |  | |  | |  | X |  | X |
|  | | | | | | | | | | | | |
| **Family history (first degree relative)** | |  |  | |  | |  | |  |  |  |  |
| MI (relative, age at occurrence) | |  | X | |  | |  | |  |  |  |  |
| Stroke (relative, age at occurrence) | |  | X | |  | |  | |  |  |  |  |
| VTE (relative, age at occurrence) | |  | X | |  | |  | |  |  |  |  |
| Diabetes (relative, age at occurrence) | |  | X | |  | |  | |  |  |  |  |
| Osteoporosis (relative, age at occurrence) | |  | X | |  | |  | |  |  |  |  |
| Hip fracture (relative, age at occurrence) | |  | X | |  | |  | |  |  |  |  |
| Cancer ( site, relative, age at occurrence) | |  | X | |  | |  | |  |  |  |  |
| Current age or age at death | |  | X | |  | |  | |  |  |  |  |
|  | | | | | | | | | | | | |
| **Gynaecological** |  | |  | |  | |  | |  |  |  |  |
| Hysterectomy status (& date) | X | | X | | X | | X | | X | X | X | X |
| Hysterectomy reason |  | | X | | X | | X | | X | X | X | X |
| Oophorectomy status (& date) | X | | X | | X | | X | | X | X | X | X |
| Endometrial ablation (& date) | X | | X | |  | | X | | X | X | X | X |
| Endometrial ablation reason |  | | X | |  | | X | | X | X | X | X |
| Fibroids (date of diagnosis) | X | | X | | X | |  | |  | X | X | X |
| Endometrial hyperplasia (date of diagnosis) | X | | X | | X | |  | |  | X | X | X |
| Endometriosis (date of diagnosis) | X | | X | | X | |  | |  | X | X | X |
| Menstrual history |  | | X | |  | |  | |  |  |  |  |
| Premenstrual Tension (symptoms, treatment) |  | | X | |  | |  | |  |  |  |  |
| Obstetric history |  | | X | |  | |  | |  |  |  |  |
| Cervical smear (latest date/result) |  | | X | | X | | X | | X | X | X | X |
| Vaginal bleeding in 6m/since last visit. (dates, severity) |  | |  | | X | | X | | X | X | X | X |
| Bleeding diary (U+ only) |  | |  | | X | | X | | X | X | X | X |
|  | | | | | | | | | | | | |
| **HRT use** |  | |  | |  | |  | |  |  |  |  |
| Use ever/since last visit | X | |  | | X | |  | |  | X | X | X |
| Date of last prescription | X | |  | |  | |  | |  |  |  |  |
| Detailed history (dates/brands) |  | | X | | X | |  | |  | X | X | X |
|  | | | | | | | | | | | | |
| **Contraception** |  | |  | |  | |  | |  |  |  |  |
| Hormone contraception ≤ 6m | X | |  | |  | |  | |  |  |  |  |
| Detailed history (with dates) |  | | X | |  | |  | |  |  |  |  |
| Sterilisation |  | | X | |  | |  | |  |  |  |  |
|  | | | | | | | | | | | | |
| **Breast disease** |  | |  | |  | |  | |  |  |  |  |
| *Invasive breast cancer ever (dates) | X | | X | | X | | X | | X | X | X | X |
| *Ca in-situ/Paget’s disease of nipple (dates) | X | | X | | X | | X | | X | X | X | X |
| *Atypical hyperplasia ever | X | | X | | X | | X | | X | X | X | X |
| *BRCA1/BRCA2 | X | | X | | X | | X | | X | X | X | X |
| Consultation for problem (reasons) | X | | X | | X | | X | | X | X |  | X |
| Excision of benign lump (date) | X | | X | | X | | X | | X | X |  | X |
| Biopsy/fine needle aspiration (date) | X | | X | | X | | X | | X | X |  | X |
| Benign disease since screening (date, diagnosis) |  | |  | |  | |  | |  | X |  | X |
| Mammography (latest date/result) |  | | X | | X | | X | | X | X | X | X |
|  | | | | | | | | | | | | |
| **Cancer** |  | |  | |  | |  | |  |  |  |  |
| *Primary site (date of diagnosis ) | X | | X | | X | | X | | X | X | X | X |
| *Cancer treatment in past 10yrs | X | | X | | X | | X | | X | X | X | X |
|  | | | | | | | | | | | | |
| **CVD** |  | |  | |  | |  | |  |  |  |  |
| Myocardial infarction (dates) | X | | X | | X | |  | |  | X | X | X |
| Atrial fibrillation | X | | X | | X | |  | |  | X |  | X |
| Angina | X | | X | |  | |  | |  | X | X | X |
| Heart failure ever |  | |  | |  | |  | |  | X |  |  |
| Stroke –TIA/CVA/sub-arachnoid (dates) | X | | X | | X | |  | |  | X | X | X |
|  | | | | | | | | | | | | |
| **Venous disease** |  | |  | |  | |  | |  |  |  |  |
| *DVT ever/since last visit | X | | X | | X | |  | |  | X | X | X |
| *PE ever/since last visit | X | | X | | X | |  | |  | X | X | X |
| *Retinal vein occlusion ever/since last visit |  | | X | | X | |  | |  | X | X | X |
| Thrombophlebitis ever/since last visit |  | | X | | X | |  | |  | X | X | X |
| Varicose veins ever |  | | X | | X | |  | |  | X | X | X |
| Varicose veins treatment (yes/no) |  | |  | | X | |  | |  | X |  |  |
| *Thrombophilia test & result |  | | X | | X | |  | |  | X | X | X |
|  | | | | | | | | | | | | |
| **Musculoskeletal** |  | |  | |  | |  | |  |  |  |  |
| Fractures ≥ 20 yrs  (max 3 with date, site and cause) ** | X | | X | |  | |  | |  |  |  |  |
| Fractures since screening/last visit  (date & site) |  | |  | |  | |  | |  | X | X | X |
| Densitometry measurement ** | X | | X | |  | |  | |  | X |  | X |
| Abnormal densitometry | X | | X | |  | |  | |  | X |  | X |
| Scoliosis/kyphosis/other spinal curvature ** |  | | X | |  | |  | |  |  |  |  |
| Low back pain (ever, past year, current) |  | |  | |  | |  | |  | X |  | X |
| Joint pain - knee, hip, hand (ACR OA criteria) |  | |  | |  | |  | |  | X |  | X |
| Joint stiffness, swelling (ever/ past yr) |  | |  | |  | |  | |  | X |  | X |
| Hip or knee injury (ever, age) |  | |  | |  | |  | |  | X |  | X |
| OA, RA, gout (age, current) |  | |  | |  | |  | |  | X |  | X |
| Current medication for arthritis |  | |  | |  | |  | |  | X |  | X |
| WOMAC (self-assessed pain, stiffness & disability) if knee or hip problem (52) |  | |  | |  | |  | |  | X |  | X |
| Hand symptom questionnaire if hand problem (53) |  | |  | |  | |  | |  | X |  | X |
| Fall in last 4m/since last visit (no.) |  | |  | |  | |  | |  | X | X | X |
|  | | | | | | | | | | | | |
| **Psycho-social** | |  | |  | |  | |  |  |  |  |  |
| *Alcohol abuse | | X | |  | |  | |  |  |  |  |  |
| *Drug abuse | | X | |  | |  | |  |  |  |  |  |
| *Schizophrenia | | X | |  | |  | |  |  |  |  |  |
| *Severe learning disability | | X | |  | |  | |  |  |  |  |  |
| CES Depression Scale  (20-item self-report depression scale) (54) | |  | |  | |  | |  |  | X |  | X |
| Cognitive function screening tests  (TICS-m & verbal fluency) (55 ) | |  | |  | |  | |  |  | X |  | X |
|  | | | | | | | | | | | | |
| **Hepato-biliary** | |  | |  | |  | |  |  |  |  |  |
| *Chronic or active liver disease | | X | |  | |  | |  |  |  |  |  |
| Liver disease ever/since last visit | |  | | X | | X | |  |  | X | X | X |
| *Hepatitis B status | |  | | X | | X | |  |  | X | X | X |
| History of gall bladder disease | | X | | X | | X | |  |  | X | X | X |
| Cholecystectomy | | X | | X | | X | |  |  | X | X | X |
| *Gall stones since cholecystectomy | | X | | X | | X | |  |  | X | X | X |
| Pancreatitis | |  | |  | |  | |  |  | X | X | X |
|  | | | | | | | | | | | | |
| **Renal** | |  | |  | |  | |  |  |  |  |  |
| *Renal impairment ≤ 3 yrs | | X | |  | |  | |  |  |  |  |  |
| Renal disease ever/since last visit | |  | | X | | X | |  |  | X | X | X |
|  | | | | | | | | | | | | |
| **SLE** | |  | |  | |  | |  |  |  |  |  |
| SLE ever w. age at Dx | |  | |  | |  | |  |  | X |  | X |
|  | | | | | | | | | | | | |
| **Asthma** | |  | |  | |  | |  |  |  |  |  |
| Asthma – ever/past year, onset, current, severity (hosp, steroids, Sx) | |  | |  | |  | |  |  | X |  | X |
|  | | | | | | | | | | | | |
| **Hearing** | |  | |  | |  | |  |  |  |  |  |
| Hearing problems & tinnitus (symptoms, severity) | |  | |  | |  | |  |  | X | X | X |
|  | | | | | | | | | | | | |
| **Incontinence** | |  | |  | |  | |  |  |  |  |  |
| Incontinence – ever/past yr (frequency, circumstances, severity, limitation) | |  | |  | |  | |  |  | X |  | X |
|  | | | | | | | | | | | | |
| **Eye** | |  | |  | |  | |  |  |  |  |  |
| Sudden loss of vision/ diplopia/ proptosis (ever/since last visit) | |  | |  | |  | |  |  | X | X | X |
| Eye disease – glaucoma, cataract, AMD, diabetic, amblyopia  (which eye / treatment / referral) | |  | |  | |  | |  |  | X |  | X |
| Dry eye symptoms | |  | |  | |  | |  |  | X |  | X |
|  | | | | | | | | | | | | |
| **Oral** | |  | |  | |  | |  |  |  |  |  |
| Tooth count | |  | |  | |  | |  |  | X |  | X |
| Dry mouth symptoms | |  | |  | |  | |  |  | X |  | X |
|  | | | | | | | | | | | | |
| **Miscellaneous** | |  | |  | |  | |  |  |  |  |  |
| Current diabetes (+/- insulin) | | X | | X | |  | |  |  | X |  |  |
| *Otosclerosis | |  | | X | | X | |  |  | X | X | X |
| *Porphyria ever | |  | | X | | X | |  |  | X | X | X |
| *Benign meningioma ever | |  | | X | | X | |  |  | X | X | X |
| Wheelchair confinement (current) | |  | | X | | X | |  |  | X | X | X |
|  | | | | | | | | | | | | |
| **Current medical conditions** | |  | |  | |  | |  |  |  |  |  |
| Up to 5 diagnoses ** | |  | | X | | X | |  |  | X | X | X |
|  | | | | | | | | | | | | |
| **Current drugs** | |  | |  | |  | |  |  |  |  |  |
| *SERMs | |  | | X | | X | |  |  | X | X | X |
|  | | | | | | | | | | | | |
| Hypoglycaemic | |  | |  | | X | |  |  | X | X | X |
| Anticoagulant | |  | |  | | X | |  |  | X | X | X |
| Thyroid hormone | |  | |  | | X | |  |  | X | X | X |
| Oral corticosteroid | |  | |  | | X | |  |  | X | X | X |
| Rifampicin | |  | |  | | X | |  |  | X | X | X |
| Phenytoin | |  | |  | | X | |  |  | X | X | X |
| TCA | |  | |  | | X | |  |  | X | X | X |
| Other (specify up to 10) | |  | |  | | X | |  |  | X | X | X |
|  | | | | | | | | | | | | |
| **Symptoms Questionnaire** | |  | |  | |  | |  |  |  |  |  |
| Menopausal, side effects (appendix 1) | |  | | X | | X | | X | X | X | X | X |
|  | | | | | | | | | | | | |
| **Quality of life** | |  | |  | |  | |  |  |  |  |  |
| Visual analogue scale (1global score) (56) | |  | | X | |  | |  |  | X | X | X |
| Women’s Health Questionnaire (subscales for mood, somatic Sx, anxiety, sexual behaviour, sleep problems, menstrual Sx) [57] | |  | |  | |  | |  |  | X |  | X |
| Self-esteem scale [58] | |  | |  | |  | |  |  | X |  | X |
| Euroquol (generic w. scores for economic evaluation) [59] | |  | |  | |  | |  |  | X |  | X |
| Schedule for the Evaluation of Individual Quality of Life (SEIQoL)[(60] ^(1)^ | |  | |  | |  | |  |  | X |  | X |
| Short Form 36 Health Survey (SF-36) [61] ^(1)^ | |  | |  | |  | |  |  | X |  | X |
|  | | | | | | | | | | | | |
| **Physical Measurements** | |  | |  | |  | |  |  |  |  |  |
| BP | |  | | X | | X | | X | X | X | X | X |
| Height ** | |  | | X | |  | |  |  | X |  | X |
| Weight ** | |  | | X | | X | | X | X | X | X | X |
| Waist & hip | |  | |  | |  | |  |  | X |  | X |
| Urine blood/protein/glucose dipstick | |  | |  | | X | |  |  | X |  | X |
| Blood for triglycerides & storage | |  | |  | |  | |  | X |  |  |  |
|  | | | | | | | | | | | | |
| **Health service use** | |  | |  | |  | |  |  |  |  |  |
| Hospitalisation in past 4m/since last visit (reason(s), length of stay(s)) | |  | |  | |  | |  |  | X | X | X |
| Out Patient visits in past 4 m/since last visit (number, reason(s)) | |  | |  | |  | |  |  | X | X | X |
| GP visits in past 4 m (no.) | |  | |  | |  | |  |  | X |  |  |
|  | |  | |  | |  | |  |  |  |  |  |
| **Trial medication** | |  | |  | |  | |  |  |  |  |  |
| Tablets (foils issued, tablets taken) | |  | |  | | X | | X | X | X | X | X |
| Temporary interruption(s) (reason(s)/duration) | |  | |  | | X | | X | X | X | X | X |
|  | | | | | | | | | | | | |
| **Participation in other studies** | |  | |  | |  | |  |  |  |  |  |
| Million Women | |  | | X | | X | |  |  | X | X | X |
| EPIC | |  | | X | | X | |  |  | X | X | X |
| Other clinical trial (current) | |  | | X | | X | |  |  | X | X | X |
|  | |  | |  | |  | |  |  |  |  |  |

*Trial exclusions

** Not collected at screening from May 2002

(1) In a sub-study only
